# Solitary intracranial osteoma with attachment to the falx: a case report

**DOI:** 10.1186/1477-7819-11-221

**Published:** 2013-09-08

**Authors:** Shu-Mei Chen, Chi-Cheng Chuang, Cheng-Hong Toh, Shih-Ming Jung, Tai-Ngar Lui

**Affiliations:** 1 Department of Neurosurgery, Taipei Medical University-Wan Fang Hospital, Taipei Medical University, Taipei 116, Taiwan; 2 Graduate Institute of Clinical Medical Sciences, College of Medicine, Chang Gung University, Kweishan, Taoyuan 333, Taiwan; 3 Department of Neurosurgery, Chang Gung Memorial Hospital at Linkou, Chang Gung University, Kweishan, Taoyuan 333, Taiwan; 4 Department of Medical Imaging and Intervention, Chang Gung Memorial Hospital at Linkou, Chang Gung University, Kweishan, Taoyuan 333, Taiwan; 5 Department of Pathology, Chang Gung Memorial Hospital at Linkou, Chang Gung University, Kweishan, Taoyuan 333, Taiwan

**Keywords:** Falx, Intracranial, Osteoma

## Abstract

**Background:**

Intracranial osteomas are uncommon lesions that usually arise from the inner table of the cranium. There are few reports in the literature of intracranial osteomas with meninges attachment and without direct relation with the skull bone; these osteomas were mostly attached with dura. We report a rare osteoma with falx attachment.

**Case:**

A 64-year-old woman presented with a 3-month history of intermittent tinnitus and dizziness. The scout film of petrous bone computed tomography scan revealed a high-density lesion in the frontal area. Magnetic resonance imaging showed a 2.5-cm mass attached to the surface of the falx in the right frontal parasagittal area. The patient underwent right frontal craniotomy, and a bony hard mass was found located in the right frontal parasagittal region extra-axially, with its medial surface attached to the falx. It could not be broken down by the cavitron ultrasonic surgical aspirator or even the cutting loop and was detached from the falx and removed in one piece. Histopathological examination showed a nodule with bony trabeculae and bone marrow tissue, compatible with osteoma. The postoperative course was uneventful, and the patient was discharged from the hospital with no neurological deficits one week after operation.

**Conclusions:**

This is the first case report in the English literature of an intracranial osteoma arising from the falx. Because of their slow growth and their locations in silent brain areas, intracranial osteomas are usually diagnosed incidentally. Surgical resection is the primary treatment choice.

## Background

Osteomas are benign neoplasms consisting of mature normal osseous tissue. They commonly arise from the long bones of the extremities. In the head and neck region, they are usually found in the sinuses, facial bones, skull, and mandible [1-9]. Intracranial osteomas are rarely located intradurally without the involvement of bony structure [10]. The mechanism that triggers the formation of intracranial intradural osteomas without bony structure involvement remains unclear. We report the clinical presentation, pathologic picture, and intra-operative findings of an intradural osteoma with attachment to the falx.

## Case presentation

**Figure 1:**
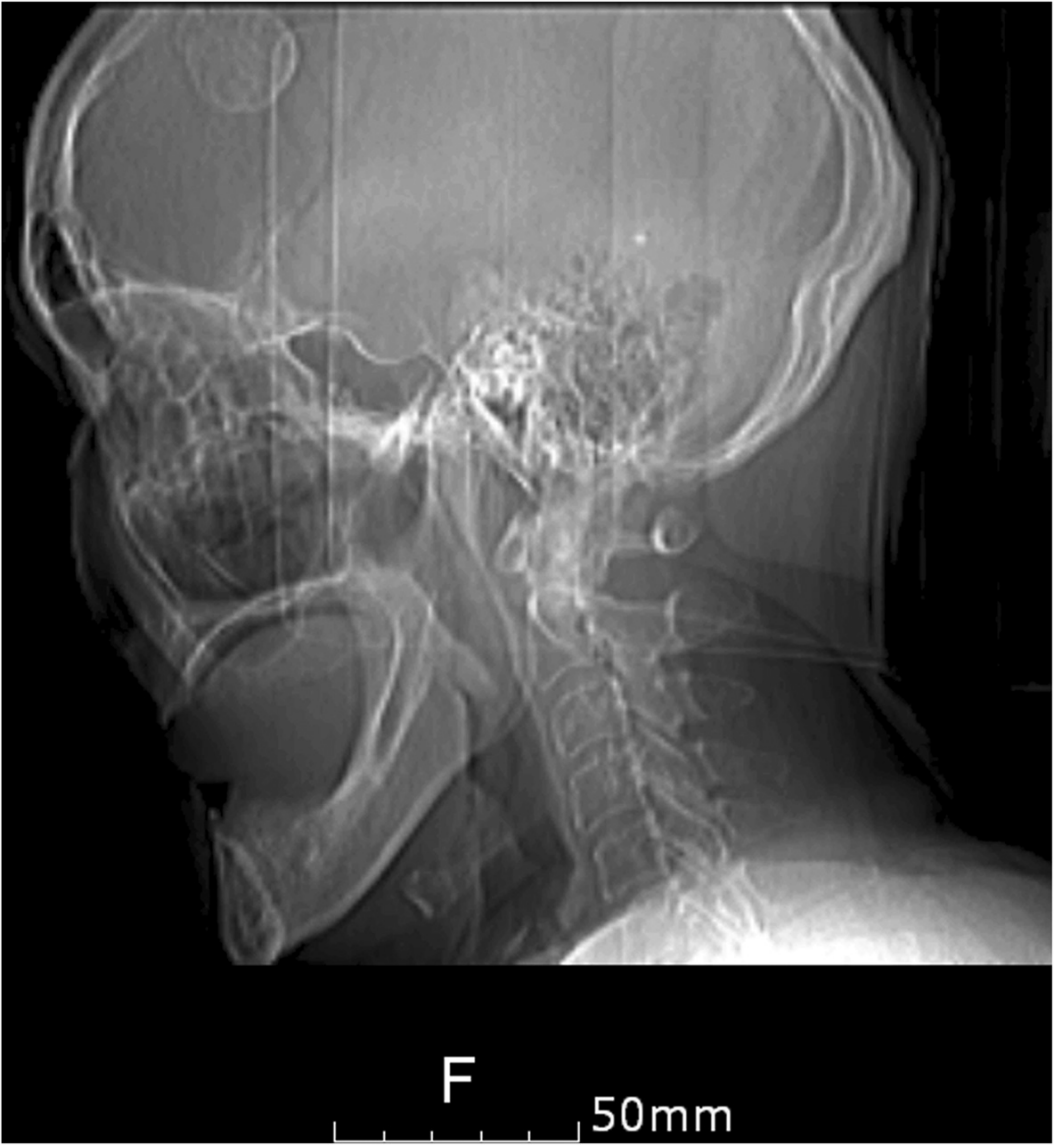
**Scout film of the petrous bone computed tomography (CT) scan.** It showed a dense radiopaque mass in the frontal area.

**Figure 2:**
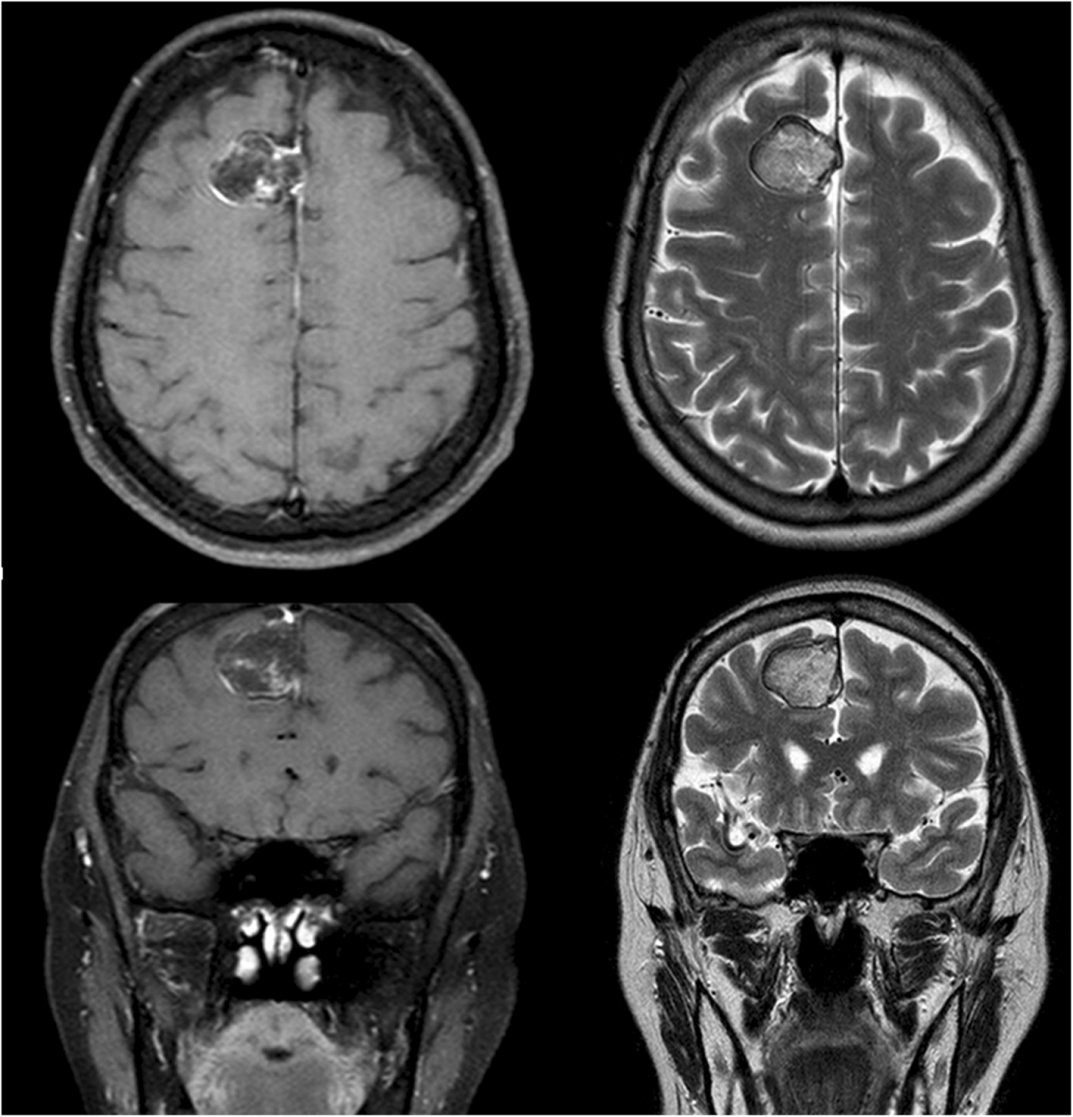
**Magnetic resonance imaging (MRI).** Axial and coronal contrast-enhanced T1-weighted images with fat saturation show a 2.5-cm falx-based mass with minimal marginal enhancement in the right parasagittal region. The mass is hyperintense with a hypointense rim on T2-weighted images.

**Figure 3:**
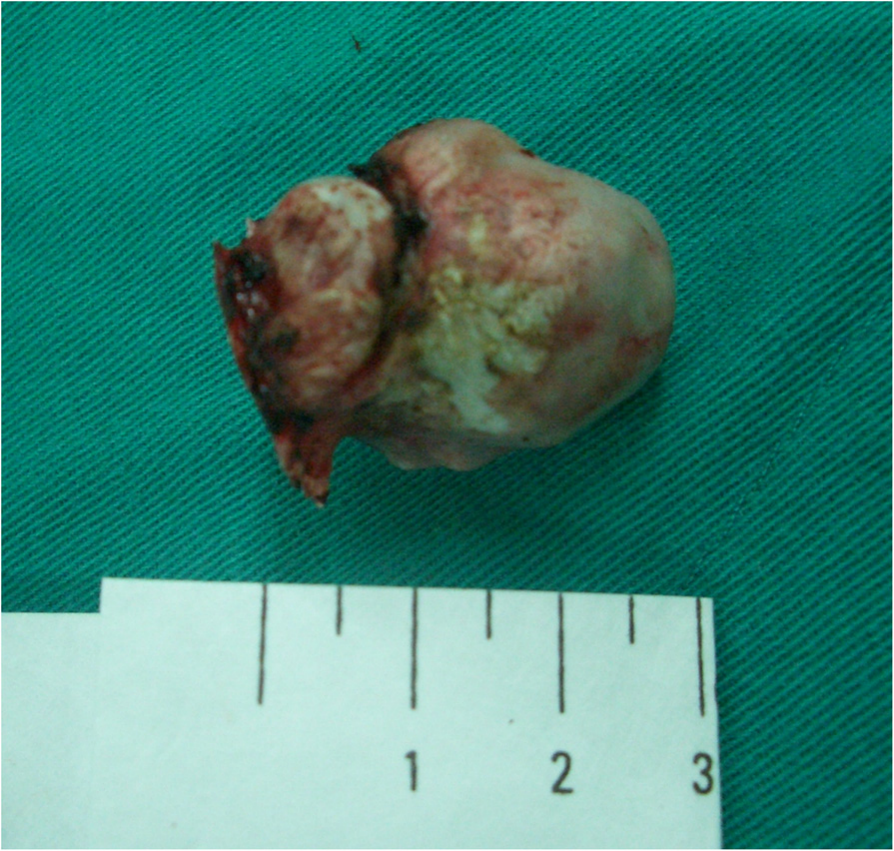
**Gross appearance of the osteoma.** The medial surface (arrow) was attached on the falx.

A 64-year-old woman presented with a 3-month history of intermittent bilateral tinnitus, occasionally accompanied by dizziness. She had no history of major head injury or systemic infection. She visited an otology clinic where a temporal bone computed tomography (CT) scan of the petrous bone, semicircular canal, and hearing apparatus showed neither temporal bone lesions nor associated lesions in the ear; however, the scout film of the CT scan incidentally revealed a radiopaque nodule in the frontal area (Figure 1). Magnetic resonance imaging (MRI) revealed a 2.5-cm mass with minimal marginal enhancement in the right parasagittal region in axial and coronal contrast-enhanced T1-weighted images. The mass was hyperintense with a hypointense rim in T2-weighted images (Figure 2). The provisional diagnosis was a calcified meningioma originating from the falx. The patient was then referred to our neurosurgical department for surgical removal of the tumor. On admission, the physical and neurological findings were unremarkable. Her cognitive function was intact, and she had no sensorimotor deficit. Right frontal craniotomy was performed. After opening of the dura and retraction of the parasagittal gyri, a tumor was found attached to the falx in the mesial frontal lobe extra-axially. The tumor was as hard as bone and could not be broken by bipolar forceps, Cavitron ultrasonic surgical aspirators, or even cutting loops. The tumor had to be dissected apart from the falx surface in one piece (Figure 3).

**Figure 4:**
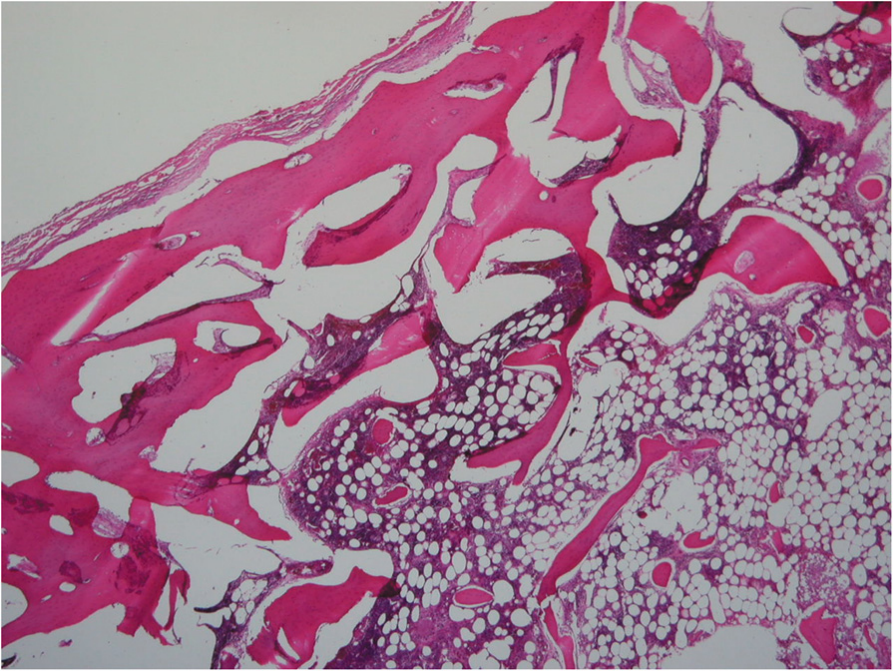
**Histopathology of the resected specimen.** Lamellated bony trabeculae and the intertrabecular space occupied by loose fibrovascular tissue as observed during pathological examination (original magnification × 20).

Pathologic examination revealed lamellated bony trabeculae lined by osteoblasts, and the intertrabecular marrow spaces were occupied by loose fibrovascular tissue (Figure 4).

The postoperative course was uneventful. The patient was discharged from the hospital with no neurological deficits after suture removal. During six months of post-operative follow-up, the frequency and degree of dizziness and tinnitus decreased progressively.

### Discussion

Osteomas are recognized as benign bone neoplasms of uncertain etiology. In the head, osteomas often arise from the periosteum of the frontal or ethmoid sinuses and the mandible [1-9]. Less often, osteomas may originate from the cranial bone in the convexity or the inner layer of the dura mater [11-14]. Osteomas attached to the meninges without relationship with the bone are even rarer. We reviewed related literature and summarized the data (Table 1). Fallon et al. [15] found intracranial meningeal osteomas in 5% of 200 adult autopsies. The tumors were usually located at the convexity dura and falx junction around the superior sagittal sinus. Solitary intracranial osteomas arising from the inner layer of the dura mater with no involvement of the cranium have been reported [11,12,14,16-18]. The tumors usually establish themselves with a wide base and grow inward as an expanding mass with a well-defined border [12]. The osteoma we report on here was a fungating nodule with a narrow neck attached to the falx.

**Table 1:** Patients with intracranial osteoma attached to the meninges without relationship with the bone reported in the literature

**No.**	**Age/Sex**	**Preoperative symptom**	**Location**	**Size**	**Surgery**	**Operative finding**	**Postoperative outcome**	**(ref.)**
1	20/F	Headache over right fronto-temporal area	Right frontal convexity	1.0 x 1.0 x 1.0 cm^3^	Right fronto-temporal craniotomy	Arose from the inner surface of dura	Asymptomatic	[12]
2	51/F	Headache	Right frontal convexity	1.1 x 1.5 x 0.7 cm^3^	Right frontal craniotomy	Partially adherent to the inner dural surface	No post-operative problem	[16]
3	28/F	Headache at left frontal area	Left frontal convexity	4.0 x 2.5 x 0.5 cm^3^	Left frontal craniotomy	Covered with arachnoid membrane	Relief of headache	[10]
4	35/M	Vertigo	Right frontal convexity	5.0 x 5.0 x 2.0 cm^3^	Right frontal craniotomy	Attached to the dura	Not available	[14]
5	24/M	Headache	Right frontal convexity	Not available*	Right frontal craniotomy	Covered with arachnoid membrane	Venous congestion post-operative 3 days	[18]
							No sign of recurrence 2 years after the surgery	
6	60/M	Headache	Right frontal convexity	Not available	Right frontal craniotomy	Attached to the dura	No neurologic deficits	[17]
7	43/F	Headache in left frontal area	Left frontal convexity	1.2 x 2.0 x 0.7 cm^3^	Left frontal craniotomy	Attached to the dura	Asymptomatic	[11]
8	64/M	Tinnitus with dizziness	Right mesial frontal lobe extra-axially	2.5 x 2.0 x 2.0 cm^3^	Right frontal craniotomy	Attached to the falx	The frequency and degree of dizziness and tinnitus decreased	Present case

* multiple.

On the basis of their natural history, intracranial osteomas are more benign than osteomas of the frontal sinuses [19]. The tumor may cause pressure-related symptoms by compressing or displacing the underlying brain, and this depends on the location of the mass. When it appears in a silent area, it rarely causes sensorimotor deficit. Most commonly, such tumors are detected in patients presenting with headache or other non-specific complaints. The high frequency of head trauma history has been noted, and the possible importance of head trauma as a causative factor in cranial osteomas has been proposed [17,20]. In our case, there was no history of major head trauma. This intracranial osteoma was found incidentally on the scout image during a temporal bone CT scan.

The pathogenesis of osteomas without bone involvement is still unknown. A dural osteoma can be confused with “meningeal ossification or meningeal calcification” originating from the dura or the falx because of the similarities in CT and MRI examinations and microscopic appearance [15]. This new bone might not represent a true neoplasm but possibly an osteogenic change in the cerebral meningeal tissue. However, meningeal ossifications are commonly multicentric and located on the dural-falx junction along the both sides of the superior sagittal sinus [21].

The factors leading to the genesis of intracranial meningeal osteomas are as yet unknown. New bone formation from the dura or falx has been postulated as one of the causes because the meninges may function as the periosteum of the inner table of the skull [10,12,15].

## Conclusions

In our patient, the operative findings confirmed an intracranial bony hard mass arising from the falx. This intradural osteoma might have been the result of a focal falx osteogenic activity. To the best of our knowledge, this is the first report of an intracranial osteoma that originated from the falx in an otherwise normal patient.

## Consent

The patient consented to the study and publication of this case report and images. A written consent is available for review.

## Abbreviations

CT: Computed tomography; MRI: Magnetic resonance imaging.

## Competing interests

The authors declare that they have no competing interests.

## Authors' contributions

SMC participated in the preparation of the manuscript, literature search and drafted the manuscript. CCC participated in its design and coordination. CHT accomplished and analysed the CT scans and MRI. SMJ carried out the tissue preparation, (immuno)histology and edited the manuscript for its scientific content. TNL was responsible for the operations, follow-up of the patients and data preparation and conceived of the study. All authors read and approved the final manuscript.
